# The Increasing Complexity of the Oncofetal *H19* Gene Locus: Functional Dissection and Therapeutic Intervention

**DOI:** 10.3390/ijms14024298

**Published:** 2013-02-21

**Authors:** Imad Matouk, Eli Raveh, Patricia Ohana, Rasha Abu Lail, Eitan Gershtain, Michal Gilon, Nathan De Groot, Abraham Czerniak, Abraham Hochberg

**Affiliations:** 1 Department of Biological Chemistry, Institute of Life Sciences, the Hebrew University of Jerusalem, Jerusalem 91904, Israel; E-Mails: eli.raveh@biocancell.com (E.R.); patricia.ohana@biocancell.com (P.O.); rusha.a.l@gmail.com (R.A.L.); eitan.gershtain@biocancell.com (E.G.); mdgilon@gmail.com (M.G.); hochberg@cc.huji.ac.il (A.H.); 2 Department of Biological Sciences, Faculty of Science and Technology, Al-Quds University, Jerusalem 51000, Israel; 3 Department of HPB Surgery “A”, Sheba Medical Center, Tel Hashomer, Tel Aviv 52621, Israel; E-Mail: abrahamc@post.tau.ac.il

**Keywords:** long non-coding RNA, transcriptional modulators, DNA-based therapy, diphtheria toxin A, sense and antisense transcripts

## Abstract

The field of the long non-coding RNA (lncRNA) is advancing rapidly. Currently, it is one of the most popular fields in the biological and medical sciences. It is becoming increasingly obvious that the majority of the human transcriptome has little or no-protein coding capacity. Historically, *H19* was the first imprinted non-coding RNA (ncRNA) transcript identified, and the *H19/IGF2* locus has served as a paradigm for the study of genomic imprinting since its discovery. In recent years, we have extensively investigated the expression of the *H19* gene in a number of human cancers and explored the role of *H19* RNA in tumor development. Here, we discuss recently published data from our group and others that provide further support for a central role of *H19* RNA in the process of tumorigenesis. Furthermore, we focus on major transcriptional modulators of the *H19* gene and discuss them in the context of the tumor-promoting activity of the *H19* RNA. Based on the pivotal role of the *H19* gene in human cancers, we have developed a DNA-based therapeutic approach for the treatment of cancers that have upregulated levels of *H19* expression. This approach uses a diphtheria toxin A (DTA) protein expressed under the regulation of the *H19* promoter to treat tumors with significant expression of *H19* RNA. In this review, we discuss the treatment of four cancer indications in human subjects using this approach, which is currently under development. This represents perhaps one of the very few examples of an existing DNA-based therapy centered on an lncRNA system. Apart from cancer, *H19* expression has been reported also in other conditions, syndromes and diseases, where deregulated imprinting at the *H19* locus was obvious in some cases and will be summarized below. Moreover, the *H19* locus proved to be much more complicated than initially thought. It houses a genomic sequence that can transcribe, yielding various transcriptional outputs, both in sense and antisense directions. The major transcriptional outputs of the *H19* locus are presented here.

## 1. The Increasing Complexity of the *H19* Gene Locus

**Figure 1:**
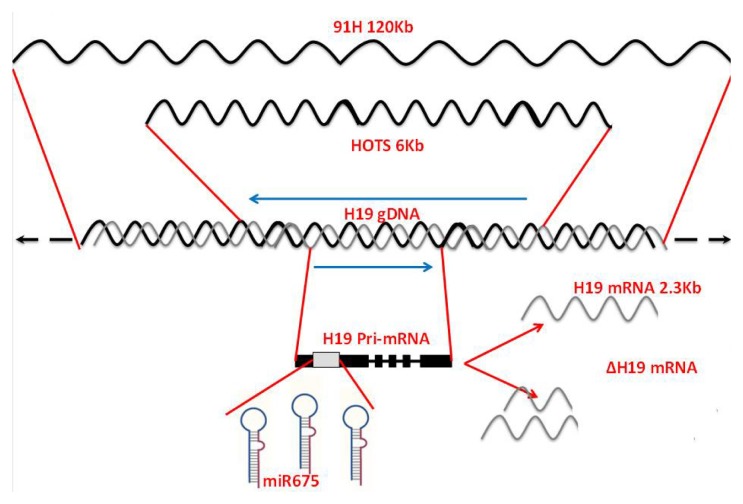
The increasing complexity of the H19 gene locus: shown is schematic representation of the *H19* gene locus showing various transcriptional products produced from both the sense and the antisense strands.

Recent efforts to better characterize the *H19* locus have revealed that this locus is housing several overlapping transcriptions on the two DNA strands that can produce different transcriptional products. The most extensively studied is H19 RNA itself, which is transcribed by RNA polymerase II and processed by capping, splicing and polyadenylation; however, it does not code for any protein product. Although the majority of the transcriptional output of the *H19* locus is non-coding RNAs, recently, it has become clear that it can also code for a protein product. In addition to *H19* itself, this locus also produces a microRNA, called *miR-675*, an antisense protein coding transcript, called *H19* opposite tumor suppressor (*HOTS*), and a long intergenic antisense transcript, called *91H* [1–3]. Moreover, long-range chromatin interactions at the mouse *IGF2/H19* locus reveal a novel paternally expressed, long non-coding RNA, called *PIHit*, which is a liver-specific capped and unpolyadenylated transcript with heterogeneous size, but it is located in a poorly characterized intergenic region between *H19* and *IGF2* and will not be discussed here [4]. Furthermore, we and others have reported the identification of an alternative splice isoforms of *H19* RNA [5,6]. Besides the *H19* gene itself, which is well characterized, we'll discuss the other products of this locus in greater detail. Figure 1 shows a schematic representation of the *H19* locus and the transcriptional products, which can be produced from it in the sense and antisense directions.

### 1.1. *H19* Opposite Tumor Suppressor (HOTS)

Transcription in both sense and antisense directions has been reported, not only for regular genes, but also for those genes that are imprinted. In the same imprinted loci of *H19*, several antisense transcripts have been reported. A transcript that is antisense to *IGF2*, IGF2-antisense (IGF2-AS), is transcribed from within *IGF2* in a reverse orientation. This transcript is also maternally imprinted [7]. *LIT1* is an antisense transcript that is normally expressed from the paternal allele and lies within the *KvLQT1* gene, and loss of imprinting (LOI) of *LIT1* is linked to abnormal methylation of a differentially methylated region (DMR) upstream from the gene [8,9]. Those are not the only examples. Antisense transcripts have also been described for the imprinted genes, *UBE3A*, *Zfp127/ZFP127*, *GNAS1 and IGF2R*, to list some examples.

The *H19* locus also produces antisense transcripts. Two such transcripts have been reported. Antisense to the *H19* gene, the human *H19* locus, encodes a translated product, called *HOTS*, for *H19* opposite tumor suppressor [2]. A long antisense transcript, named *91H* RNA, was also reported for both the human and the mouse *H19/IGF2* loci [3].

Further characterization of *HOTS* indicated that it is an imprinted transcript, with the maternal allele actively expressed. *HOTS* transcript extends 1 kb upstream and 2.8 kb downstream of the *H19* gene, comprising an approximate 6 kb polyadenylated transcript and containing a CpG island promoter [2].

The *HOTS* transcript is associated with the polysomes, suggesting its translation. The longest open reading frame (ORF) contained in *HOTS* that reside entirely on the *H19* transcriptional unit encodes a predicted polypeptide of 150 amino acids that is well-conserved in primates, but not in the mouse, which has no ORF. A polyclonal antibody raised against *HOTS* detects *HOTS* in fetal tissues in monomeric and dimeric states and also in differentially expressed isoforms. Furthermore, *HOTS* has a putative nuclear localization signal and resides in the nucleus and the nucleolus, where it co-localizes with nucleophosmin [2].

Regarding the function of *HOTS*, data support a tumor suppressor activity. Using both overexpression and knockdown approaches, potent growth inhibition *in vitro* was observed and also a decrease in the ability of these cells to form colonies in soft agar. Furthermore, the *in vivo* tumorigenic potential of those cells was significantly impaired [2].

However, functioning as a tumor suppressor should at least indicate a possible mutation or downregulation of *HOTS* in cancer patients. To answer this question, Onyango *et al*. tried to identify mutations within the coding region of *HOTS* in samples of Wilm’s tumors. No such mutations were detected. However, analyses of a few cases of Wilm’s tumor with loss of heterozygosity (LOH) at *11p15* showed loss of *HOTS* expression [2]. It is clear that further work will be needed to understand the complex regulation of *HOTS* and *H19* expression and the interrelationship between them and within the imprinted cluster as a whole.

### 1.2. Identification of *91H*: A Long Intergenic Antisense RNA

A novel long intergenic 120-kb transcript antisense to the *H19* gene has been identified at both the human and the mouse *H19/IGF2* imprinted loci and is called *91H*. The *91H* RNA is short lived and almost exclusively nuclear, contrary to *H19*, which has equal distribution in the cytoplasm and the nucleus. In humans, the 5′ limit is located within intron 1 of the *MRPL23* gene, located about 40 kb downstream of *H19*, and the 3′ limit is located about 70 kb upstream of *H19*. Thus, its promoter lies in an intron of an active host mRNA gene (*MRPL23*) [3].

Furthermore, it was shown that *91H* was strongly and systematically over-expressed in breast cancer compared to normal breast tissues. The *91H* RNA accumulation in breast cancer cells likely results from transcript stabilization. Like *H19*, the *91H* RNA is also monoallelically expressed from the maternal allele [3].

The expression of *91H* is very similar to *H19*, being dependent on the developmental stage in its regulation. It is also induced by the process of myoblastic cell differentiation, which may indicate that both *H19* and *91H* are sharing common regulatory schemes, which allow gene transcription in a time-restricted manner.

The functional role of *91H* is addressed using RNAI technology. Although *91H* silencing does not modify *H19* imprinting status and has a minimal effect on *H19* expression, it strongly reduces *IGF2* mRNA levels.

It is not clear yet if *HOTS* forms a part of *91H*.

### 1.3. *H19* Is a Primary MicroRNA Precursor

One way by which ncRNA may acquire a function is by being a precursor of microRNA, which is capable of performing a regulatory function. Indeed, produced from exon 1 of *H19*, a miRNA-containing hairpin serves as a template for two distinct miRNAs, *miR-675-5p* and *miR-675-3p* [1]. Both, the full length *H19* RNA and its miRNA have been identified also in marsupials. The stem loop region within *H19* RNA is a highly conserved feature of the *H19* RNA, clearly demonstrating that selective pressure may be higher on the miRNAs region than on *H19* as a whole during mammalian evolution [10]. Apart from imprinting regulation, this important finding indicates that *H19* may also control gene expression by post-transcriptional mechanisms. Whether *H19* and its *miR-675* have distinct functions or whether *H19* functions through its *miR-675* remains to be elucidated.

Recently, it was observed that no coordinated expression of *H19* and *miR-675* could be elucidated in tissues that are known to express a high level of *H19* RNA during all stages of embryonic development, including the liver, while expressing only low levels of *miR-675*. This suggests the existence of an inhibitory mechanism hindering the processing of *miR-675* from *H19* RNA. However, in the placenta, maternal *miR-675* expression is detected with increasing abundance from E11.5 till term [11].

Using a variety of approaches, the protein inhibiting *miR-675* processing at least in part was identified as the RNA-binding protein HuR. HuR binds the full-length *H19* RNA and inhibits the processing of *miR-675*, probably at the Drosha step. Furthermore and using an *H19* mouse model carrying a 3-kb deletion of just the *H19* transcription unit, including *miR-675* to exclude the effect of *IGF2*, it was shown that placental overgrowth results, implying that *miR-675* is a negative regulator of placental size. Apart from regulating the abundance of *IGF2* through imprinting, the *H19* locus can also regulate *IGF2* abundance through targeting its receptor, IGF1R, by *miR-675*. Increased levels of *miR-675* in the placenta coincide with downregulation of IGF1R that may contribute, at least in part, to the growth inhibiting effect on the placental size [11].

*miR-675* has been assigned a contradictory role in the process of proliferation, and it seems to function differently in normal *versus* cancer cells. Under normal conditions, *miR-675* inhibits human trophoblast cell proliferation by directly downregulating Nodal Modulator 1 (NOMO1) protein expression, binding to the 3′-UTR sequence of NOMO-1. NOMO1 is an antagonist of Nodal, which could attenuate Nodal signaling [12]. Moreover, it was shown that *miR-675* slows cell proliferation and, thus, can limit placental overgrowth, as discussed above. However, both *H19* and *miR-675* were found to be upregulated in human colon cancer, both in cell lines and primary colorectal cancer biopsies relative to adjacent non-cancerous tissues. Moreover, an inverse correlation was observed between the expression of retinoblastoma (RB) and *H19*/*miR-675*. Through both over-expression and knockdown approaches, the oncogenic property of *miR*-*675* was revealed. Suppression of *miR-675* decreased cell growth and soft agar colony formation in human colon cancer cells, while overexpression increased cell growth and soft agar colony formation. This phenotypic effect of *miR-675* could be performed at least in part by targeting the tumor suppressor, RB [13].

A lot remains to be performed, mainly in the identification of the *miR-675* targets and understanding its regulation and function. In adult human chondrocytes, expression of *H19* (and subsequently *miR-675*) is driven by *SOX9* to positively regulate *COL2A1*, which encodes a collagen protein important to the cartilage matrix [14].

### 1.4. Alternative Splice Isoforms of the *H19* Gene

We have reported the identification of an alternative splice isoform of *H19* RNA that lacks part of exon 1. This variant was detected in human embryonic and placental tissues, but not in bladder or hepatocellular carcinomas. A very low level of this variant was also detected in colon carcinoma. The observed pattern of expression suggests that this splice variant is developmentally regulated and that it plays a role in normal embryonic development, rather than in oncogenesis [5].

In principle, a minor transcript, such as the one described here, could function in several different ways to produce a biological phenotype. The change in the secondary structure resulting from splicing, as deduced from computational analysis, could indicate a special role for this variant in a certain developmental window. Another possibility is that its activity resides in the sequences at the splice points embedded within the primary transcript, rather than in the mature mRNA itself [5].

Another possibility is that this alternative spliced variant affects HOTS protein or *miR-675* processing.

An additional alternative splice variant of H19 has been reported. This variant transcript, which lacks exon 4, is either not found at all, is widely expressed or is confined to extra-villous cytotrophoblasts in first trimester placenta, depending on a combination of the genotype and the sex of the transmitting parent. This variant is genotype-specific [6].

## 2. Transcriptional Control of the Oncofetal *H19* Gene: Interplay of Factors Critical for the Tumorigenic Process

Although the role of protein coding genes in the tumorigenic process has been extensively investigated, the involvement of non-coding genes in this process is less well characterized. Like protein-coding genes, ncRNAs show complex patterns of tissue specific expression that, combined with their capacity to regulate many targets, are crucial for developmental, physiological and pathological decisions.

Rather than being one of the highest expressed genes in embryogenesis and placental development, *H19* is either highly expressed and/or manifests an aberrant allelic pattern of expression in most types of human cancer with marginal postnatal expression in most tissues. Regarding its role in tumorigenicity, it should be mentioned that although the prevailing view is that *H19* is behaving like an oncogene, yet, several groups reported *H19* as possessing a tumor suppressor activity. The *H19* locus harbors the potential to produce various products, as discussed above, with different functions, and this may account for this discrepancy. Moreover, *H19* tumorigenic activity may depend on p53 status, as proposed by us earlier and reviewed elsewhere [15–18].

In recent years, it has become increasingly clear that *H19* gene expression is essential for human tumor growth and is regulated by a complex interplay of malfunctioning factors that are reported to play a critical role in tumorigenesis. As more transcriptional modulators of H19 are discovered, *H19* regulation and function will also be better understood. In this section, we'll focus on four transcriptional modulators of the *H19* gene and discuss them in the context of the tumor-promoting activity of *H19* RNA.

### 2.1. The Oncofetal *H19* RNA Connection: Hypoxia, p53, HIF1-α and Cancer

Every solid tumor encounters hypoxic regions when grown beyond certain diameters. Hypoxia is considered a major trigger for a number of pathways that have substantial impacts on tumor progression affecting tumor angiogenesis, metastasis and chemo-resistance. It is also associated with poor prognosis, at least in some types of human cancers. All of these conditions are associated with *H19* RNA upregulation [15–18]. As hypoxia readily occurs in the majority of solid tumors, driving critical steps in tumor development and metastasis, it is of great interest to identify signaling pathways involved in this fatal outcome. In our recent studies, we identified hypoxia as a key trigger that activates the expression of the *H19* gene, and we also uncovered a major transcriptional interplay between both positive and negative regulators dictating *H19* expression under hypoxic stress. Furthermore, *H19* downstream targets have been identified using both overexpression and knockdown approaches [17,18].

Following our initial study showing *H19* induction upon hypoxia in certain cell lines, we screened about thirty different carcinoma cell lines of different lineages and origins for their ability to induce *H19* RNA in hypoxic stress [18]. Cells respond differently to hypoxic stress in terms of *H19* modulation. To gain insight into the possible mechanism associated with the *H19* response to hypoxia, we searched for a common denominator among those cell lines that do not upregulate *H19* upon hypoxia.

HIF-1 and p53 are two of the most intensively studied transcription factors; both factors are involved with hypoxic stress adaptation and are responsible, at least in part, for the above listed phenotypes of the tumor triggered by hypoxic stress. Both factors are maintained at low or undetectable levels within non-stressed cells, but are rapidly stabilized as a result of post-translational modifications, hydroxylation of HIF-1 and phosphorylation of p53. HIF-1 signaling is critical for mediating cellular adaptation to hypoxia, whereas p53 promotes hypoxia-induced apoptosis. The outcome of this interplay has major consequences on tumor progression.

We recently demonstrated a tight correlation between *H19* RNA elevation by hypoxia and the status of p53. Cells harboring wild-type p53 (p53^wt^) do not induce *H19* RNA in their response to hypoxia, whereas in cells carrying a mutated p53 (p53^mt^), the *H19* message is significantly induced, most strongly in p53-null cells.

Additionally, through various approaches, we identified HIF1-α as the factor responsible for *H19* elevation under hypoxic stress [18].

In light of our study, a molecular mechanism integrating *H19*, p53 and HIF1-α to hypoxic stress response is uncovered. In p53^wt^ cells, simultaneous suppression of p53 and overexpression of HIF1-α are needed to induce H19 significantly, while each treatment separately results in a mild induction, indicating that the molecular mechanism of p53 suppression on *H19* may, at least in part, involve interfering with HIF1-α activity [18].

**Figure 2:**
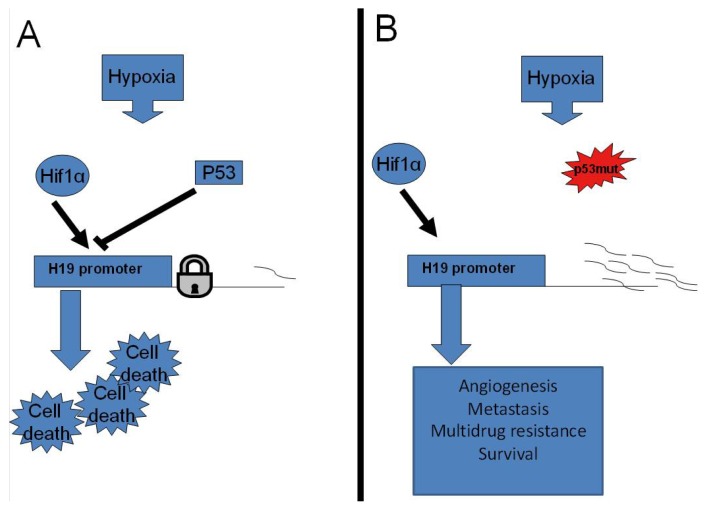
The oncofetal H19 RNA connection: hypoxia, P53 and cancer. (**A**) Under hypoxic conditions, HIF1-α upregulates *H19* RNA expression in p53 mutant cells. *H19* induction under hypoxic stress thus contributes to various aspects of tumor progression, including survival, angiogenesis, metastasis and also multidrug resistance; (**B**) P53 wild-type suppresses *H19* RNA upregulation by HIF1-α, probably leading to cell death.

Our findings suggest that H19 induction upon hypoxia results from loss of p53 function and HIF1-α increased activity observed in a large variety of human cancers (Figure 2). Our earlier observation that *H19* modulates genes whose expression is functionally involved in angiogenesis, survival and tumorigenesis in hypoxic stress indicates that *H19* RNA could grant a selective advantage to tumor growth under stress conditions [2,12], which are important phenotypes resulting from p53 loss of function and HIF1-α increased transcriptional activity (Figure 2).

In accordance with this, several lines of evidence support the involvement of *H19* RNA in tumor progression through the metastatic pathway triggered by hypoxic stress. *H19* RNA alters expression profiles of genes involved in metastasis and blood vessel development, supporting the notion of a role for this gene in tumor invasion and angiogenesis. Moreover, *H19* is highly expressed in a large proportion of hepatic metastases tested. The level of *H19* expression was also strongly correlated with tumor invasion of the reproductive organs and significantly correlated with neoplastic cell invasion of the myometrium. Moreover, the *H19* gene is expressed within both epithelial and stromal components of human invasive adenocarcinomas, where high expression matches tumor invasion.

When epithelial cells acquire mesenchymal, fibroblast-like properties and show reduced intercellular adhesion and increased motility, this process is termed epithelial-to-mesenchymal-transition (EMT). EMT is critical for embryonic development in multicellular organisms. Moreover, EMT is a potential mechanism for carcinoma progression. Our preliminary results linked *H19* upregulation to the process of EMT in carcinogenesis and embryogenesis. Induction of EMT in cancer cells by different approaches (e.g., hypoxia, TGFβ) is accompanied by *H19* upregulation.

### 2.2. *c-Myc* Oncogene Directly Induces *H19* to Potentiate Tumorigenesis

*c-Myc* is one of the transforming members of the *Myc* family and functions as a transcription factor. It obligatorily heterodimerizes its partner, *Max*, to bind DNA sequence elements, called E-boxes, and uses various mechanisms to allow target gene transcription. Numerous gene targets have been identified for *c-Myc*, including those functioning in proliferation, apoptosis, transformation and angiogenesis, however, little is known regarding the transcriptional target(s) of *c-Myc* responsible for its strong transforming abilities.

One of the transcriptional targets of *c-Myc* is *H19* non-coding RNA. A strong association between *H19* and *c-Myc* expressions was evident in both primary breast and lung cancer biopsies. *c-Myc* strongly induces the expression of *H19* and binds directly to unmethylated E-boxes close to the imprinting control region. *c-Myc* specifically binds and regulates the active maternal allele, but not the silenced paternal allele. It was shown that the histone acetylation activity is recruited by *c-Myc* to the *H19* promoter, leading to its activation without affecting the imprinting of the *H19/IGF2* locus [19].

Given the strong association of *H19* and *Myc* expression, the essential role of *H19* in transformation suggests that *Myc*-induced *H19* expression contributes to tumor etiology and the strong oncogenic behavior of *Myc* [19].

### 2.3. E2F Activates *H19* Promoter

*E2F* transcriptional regulators control human-cell proliferation by repressing and activating the transcription of genes required for cell-cycle progression, particularly the S-phase. Their associations and binding to *E2F*-responsive promoters are cell-cycle selective. The first member of this protein family to be cloned is the E2F1 and is largely considered to be the critical factor for G1/S-phase transition through the regulation of the transcription of several gene products needed for DNA synthesis. Furthermore, E2F1 is involved in tumorigenesis of several types of human cancers.

Our previous works have shown that overexpression of the *H19* gene can override cell growth arrest and induce cells to enter the S-phase in serum starved conditions accompanied by suppression of the expression of *p57Kip2* [20]. This is consistent with the results obtained from other group showing that *H19* RNA is important for entry into the S-phase after recovery from serum starvation by *E2F* binding to its promoter [21]. Moreover, *H19* is suppressed by the tumor suppressor p53 [22]. As discussed above, *miR-675* targets the tumor suppressor, RB. All of these observations place *H19* in the core of cell cycle control especially in the transition from G1- to S-phase.

A correlation between *H19* and *E2F1* expression levels in breast cancer was also revealed. Both genes are weakly expressed in normal breast cells, but upregulated in breast cancer [21].

However and through overexpression and knockdown approaches, we cannot detect a significant effect for *H19* on cell proliferation under normal cell growth conditions [20]. Under stress conditions of serum starvation and hypoxia, *H19* confers a clear growth advantage. Hypoxia and serum starvation are both the consequences of a poorly vascularized tumor, which is considered a normal stage during tumor development. Our results indicate the importance of *H19* gene expression for the tumor under stress conditions, induced by poor vascularization and characterized by poor oxygen and growth factors availability.

We previously hypothesized on the possible role played by *H19* RNA in tumorigenicity. The microenvironment of the tumor is the factor that imposes the selective pressure on the malignant cells. *H19* expression, occurring in a malignant cell and possibly enhancing its ability to proliferate under harsh condition, will give that cell a selective advantage. The progeny of the *H19*-expressing cells will increase in number at a higher rate than the neighboring cells and, hence, gradually increase their relative contribution to the tumor volume [15].

Phenotypic traits beneficial for cell proliferation also seem to favor invasive growth and metastasis. In stressed condition, those cells that manage to proliferate will indeed contribute greatly to the generation of a patho-physiological microenvironment with low extracellular pH (more acidic) and low oxygen tensions (hypoxia). Experimental studies have shown that hypoxic tumors may also be pro-angiogenic and pro-metastatic. Hypoxic conditions in tumors induce the release of cytokines that promote vascularization and, thereby, enhance tumor growth and metastasis.

Since we provide evidence that *H19* RNA enables the cells to continue proliferating in a serum starved and hypoxic condition (one of the consequences of a poorly vascularized tumor), it is logical to assume that the relative contribution of *H19* expressing cells towards the creation of the pathological microenvironment is great, which will promote tumor angiogenesis, invasion and metastasis.

## 3. *H19* Gene Expression in Non-Cancerous Diseases

*H19* gene expression was reported in several specific physiological conditions and non-cancerous disease states. We previously hypothesized that *H19* comprises a corner stone of the embryonic molecular circuit that cancer cells call upon, which otherwise remain quiescent and are under strict control, but are activated under specific conditions [16]. In some cases, it seems obvious that the *H19* gene activation in cancer and non-cancerous states share a common denominator in terms of the triggers and the targets [16].

Following are some cases, disorders and syndromes where *H19* RNA is modulated.

### 3.1. Beckwith–Wiedemann Syndrome

Beckwith*–*Wiedemann syndrome (BWS), a somatic overgrowth disorder with predisposition to embryonic tumors, is known to be caused by various epigenetic and genetic alterations, which affect regulation of the imprinted genes located on chromosome *11p15.5* [23]. The imprinting center 1 (ICR) that resides between the *IGF2* and *H19* loci is normally differentially methylated, leading to *IGF2* expression from the paternal chromosome together with silencing of *H19* expression. However, in 2%–7% of BWS patients, both alleles are methylated, leading to *IGF2* loss of imprinting and loss of *H19* expression (*H19* epimutation). This gain of methylation leads also to predisposition to develop Wilm’s tumor, hepatoblastoma and other childhood tumors [23]. However, *IGF2*, rather than *H19*, is the key factor in the development of BWS, since in many cases of BWS, normal methylation and expression of *H19* is accompanied by *IGF2* bi-allelic expression [24].

### 3.2. Russell—Silver Syndrome

Russell*—*Silver syndrome (RSS) is a heterogeneous disorder associated with growth restriction (both pre- and post-natally) and relative macrocephaly. As opposed to its reported hypermethylated status in BWS, ICR1 on chromosome *11p15.5* is significantly hypomethylated in ~20%–60% of RSS patients [25]. Regarding the complementary phenotypes of RSS and BWS, lack of *IGF2* due to epimutation, rather than *H19* bi-allelic expression, forms the basis of this syndrome, although a transgenic mouse model overexpressing *H19* seems to suggest that *H19* transcription levels have a trans-effect on gene imprinting in the embryo and on *IGF2* levels [26]. However, one study does suggest a functional role for *H19* transcripts in RSS etiology [27]. In this study, a cohort of 44 SRS patients was screened for variations in the transcribed region of *H19*. In two of the patients, different single 3 bp deletions, confined to exon1, were identified. In a third patient, 39 bp duplication was identified across exon 2 and intron 2. In two of the three patients, an abnormal *H19* splicing pattern was detected by *in vitro* studies. However, lymphocytes analysis of one of these two patients carrying the duplication did not verify an altered expression pattern of *H19*. Thus, it is possible that *H19* transcripts have a role in SRS etiology—either as an epigenetic factor or as an active noncoding RNA with altered functions.

### 3.3. Obesity, Overweight and *H19*

The connection of the *H19* locus to growth disorders may suggest a possible involvement of the *H19* locus in being overweight and obesity. Indeed, it was reported that one-year-old children who were overweight or obese had significantly higher methylation percentages at the *H19* DMR at birth compared with children who were not overweight or obese [28]. Moreover, it seems that, as a response to starvation during periconception, *ICR1* becomes hypermethylated, leading to restricted growth due to lower *IGF2* expression in adult life. Individuals who experienced famine prenatally during the second world war had hypomethylated *IGF2* DMR 60 years later, compared with their same-sex siblings that did not experience hunger [29]. *H19* DMR was not significantly associated with exposure to hunger in those individuals; however, the implications of the methylation profile reported on *H19* expression levels in the cohort above were not detected. Apparently, also here, *IGF2* is the key factor in the process.

### 3.4. Inflammation, Rheumatoid Arthritis and *H19*

Since inflammation, especially chronic, can cause cancer [30] and since *H19* is a major player in many cancer types, the question arises whether *H19* regulation/expression may change during the inflammatory process. One model that was thoroughly examined for *H19* expression in this context is rheumatoid arthritis (RA) [31], an inflammatory disease resembling cancer in many features, like, for example, the metastatic-like invasiveness of the synovial tissue into cartilage and bone, expression of several proto-oncogenes and mutations in p53. As mentioned above, *H19* is upregulated in hypoxic cancerous cells, as well as upon starvation stress and reacts to several stress related factors, such as thioredoxin, MMK1, NF-Kb, JNK2, TNF-α and IL-6. The synovial tissue in RA is also under oxidative stress. Comparing RA synovial tissue to osteoarthritis and normal/joint trauma controls revealed a higher expression of *H19* transcripts both *in situ* and by semi-quantitative PCR. RA synovial tissue-derived cultured fibroblasts elevate their *H19* level following starvation, as compared to the control cultures mentioned above, in which starvation needed an additional stimulation, such as IL-1β or PDGF-BB. However, since the change observed was mainly quantitative rather than qualitative, *H19* cannot be regarded as a tumor marker in RA, since it is also expressed in chronic inflammatory diseases, such as osteoarthritis and normal/joint trauma However, *H19* may support dedifferentiation of the RA synovial tissue and a risk for tumor development. The correlation between this risk, which indeed exists for several cancers [32,33] and *H19* levels in the RA synovial tissue is yet to be tested. It is worth noting, however, that in a study looking at 100 patients, several tumor markers were found to be elevated in RA patients’ serum without any predictive value for tumor development [34]. Hence, *H19* elevation in RA may reflect only the inflammatory stress, without further interpretations.

### 3.5. *H19* Involvement in Pre-Eclampsia

Pre-eclampsia (PE) is a gestational hypertension, which affects 2%*–*7% of pregnancies (threatening both, mother and fetus lives) worldwide and is considered as the most common dangerous complication of pregnancy. Its symptoms disappear following the removal of the placenta and, hence, is regarded as a placental disease [35]. Since imprinting is a placenta-related process and *H19* null mice display hyperplasia of all layers of the placenta [36], *H19* involvement in its pathology is not surprising. Screening of women’s placentas has revealed *H19* LOI in 46.15% of pre-eclamptic patients (disregarding the chronologic onset of the disease), but no LOI in the healthy control group. Moreover, in the pre-eclamptic group, severe clinical symptoms (blood pressure and urine protein) were observed in the biallelic expressing group, compared to patients with monoallelic expression [37]. However, the status of *H19* imprinting in these patients was not correlated with *H19* transcription levels, which did not increase in the biallelic expressing group. This observation may be explained by post-transcriptional, alternative regulation levels of *H19* transcript levels, which obviate the regulation by imprinting. Additionally, *miR-675* levels were not examined in this study, although they may differ between the groups in the study. It is worth noting that in a French study, in which villous cytotrophoblasts were isolated from PE patients and compared to control cells from healthy patients grown under normoxic or hypoxic conditions, it was found that many of the hypoxia induced genes were also induced in PE (which may reflect the hypoxic stress during improper placentation in PE). Among the genes induced in both healthy hypoxic cells and PE cells was *H19*, which, as mentioned above, is known to be induced by hypoxia [38].

Nevertheless, the nature of the relevance of *H19* to PE is still controversial. In a Canadian study of PE patients that screened for methylation levels in *H19/IGF2* ICR1, no differences between healthy control placentas and PE placentas were observed [39]. However, other studies, also in the Chinese population [40], showed that global methylation, together with DNA methyltransferase 1 transcription levels, were increased in placentas with early onset of PE. *H19* promoter methylation levels were also increased within these placentas, and this methylation status was also expressed in reduced transcription levels of *H19*. Since *H19* is mostly expressed in the invasive intermediate trophoblasts [41] and villous cytotrophoblasts, but abundant in syncytiotrophoblasts [42,43], it may be regarded as an invasion-promoting factor in trophoblasts, possibly supporting proper placentation. Further to this study, *in vitro* assays in trophoblasts cultures revealed that *H19* has anti-proliferation effects in culture, mediated by *miR-675* [12]. Deeper analysis showed that *miR-675* levels decrease in patients with early PE onset, and this decrease was suggested to mediate an increase in NOMO1 protein levels, a suppressor of the anti-proliferative and pro-apoptotic Nodal pathway [12].

In conclusion, observations concerning *H19* expression and function in PE etiology should be standardized before it may (or may not) support usage of *H19* as a biomarker for estimating the risk of developing PE [44].

## 4. Therapeutic Applications Based on *H19* Gene

*BC-819* (also known as *DTA-H19*) is a double stranded DNA vector carrying the gene for diphtheria toxin A (DTA). *BC-819* has been designed as a targeted cancer therapy to selectively kill tumor cells. Selective tumor cell destruction is mediated by the production of DTA, a strong inhibitor of protein synthesis, thereby causing cell growth arrest. This discrimination is achieved by controlling the expression of the DTA gene with the *H19* gene promoter. Use of the *H19* promoter restricts DTA gene expression to cells expressing the H19 gene. While the *H19* gene is not expressed in healthy adult cells, it is expressed in a wide variety of tumor cells of different origins. Thus, *BC-819* has the potential to treat several types of cancer, provided the involved tumor cells express the *H19* gene, while sparing healthy cells from the DTA-mediated toxicity.

This treatment is therefore being paired with tumor tissue screening for the presence of *H19* ribonucleic acid (RNA). Patients are eligible for the treatment with *BC-819* only if their tumor is positive for *H19* RNA. This prerequisite ensures treatment of the appropriate patient population and, thus, enhances the probability of success of the proposed therapy.

Treatment of four indications is currently under development:

- transitional cell carcinoma (TCC) of the bladder;
- pancreatic cancer;
- ovarian cancer; and
- hepatic metastases

For TCC of the bladder, *BC-819* is administered intravesically after mixing with the cationic transfection agent polyethyleneimine (PEI) (*in vivo*-jetPEI™) to enhance its *in vivo* transfection efficiency.

For the other indications, *BC-819* is given by local-regional routes of delivery (intratumorally, intraperitoneally (IP) or by hepatic artery infusion (HAI)) without PEI.

These four indications were selected because (i) tumor specimens showed high levels of expression of *H19* in the majority of tissues tested from each type of tumor; (ii) animal models supported efficacy in each of these tumors and (iii) each of these malignancies is accessible for loco-regional administration of *BC-819*.

### 4.1. Bladder Cancer

In superficial transitional cell carcinoma of the bladder, a Phase 1/2a dose escalation, safety and preliminary efficacy study was completed and a Phase 2b clinical trial has completed accrual under a US Investigational New Drug (IND) application.

The phase 1 study enrolled patients with recurrent superficial TCC of the bladder, which had relapsed after prior BCG therapy and expressed *H19* RNA. A total of 18 patients were treated with intravesical *BC-819* at doses of 2–20 mg weekly and could receive periodic maintenance therapy for up to one year. This was a heavily pre-treated patient population.

Median time to recurrence among all 18 patients was 5.7 months (95% CI 4.1*–*11.6). One-year recurrence-free survival was approximately 30%. Treatment was well-tolerated. No severe toxicity was observed and no deaths occurred during the study or follow-up period. The most frequently reported adverse events (AEs) considered at least possibly related to investigational product for any dose cohort were mild to moderate in severity and were most commonly renal and urinary disorders. Therefore, the dose of 20 mg was selected for the Phase 2b study [45].

A Phase 2b study multicenter trial of intravesical *BC-819*/PEI in patients with intermediate-risk superficial bladder cancer is being completed in patients with histologically confirmed recurrent superficial bladder cancer who failed prior therapy with BCG and chemotherapy. Patients with carcinoma *in situ* were excluded. Patients received an induction phase of six weekly treatments, followed by a safety and efficacy assessment at week 9. Subsequently, in the absence of recurrence or toxicity necessitating discontinuation of therapy, patients received three three-week courses of *BC-819*/PEI maintenance therapy every 12 weeks for up to one year.

A total of 47 patients were recruited, 39 formed the per-protocol population, eight treated patients were not evaluable due to various protocol violations and were replaced. Among the first cohort, 9/18 patients (50%) had complete resolution of the target lesion at visit 7 (week 8*–*10). For all patients entered, three-months and one-year recurrence-free survivals are 63% and 48%, respectively.

Treatment has been well-tolerated. No patients have discontinued due to treatment-related AEs. The most commonly reported AEs were urinary tract disorders, such as urinary frequency and dysuria, in 34% of patients treated, all of which were mild or moderate. No treatment-related serious adverse events have been reported, except one case of possibly related hematuria.

### 4.2. Pancreatic Cancer

A Phase 1/2a dose escalation safety and preliminary efficacy for the treatment of patients with locally advanced unresectable pancreatic cancer was completed.

In a phase 1 study, nine patients with unresectable locally advanced pancreatic carcinoma (LAPC) received four doses each of intratumoral *BC-819* at either 4 or 8 mg per dose. Treatment was well-tolerated with no treatment-related AEs, and the maximum tolerated dose (MTD) was not reached. Three patients in the 8 mg cohort demonstrated partial response (PR) at their three-month assessment [46].

A phase 2b study testing higher doses of *BC-819* in combination with gemcitabine, the standard drug used, is currently ongoing in Israel and in the US to assess efficacy and safety in locally advanced, unresectable pancreatic adenocarcinoma. Patients received gemcitabine weekly for four weeks and were then randomized to 8 or 12 mg of intratumoral *BC-819* administered via endoscopic ultrasound for a core treatment of seven administrations of *BC-819* together with six additional infusions of gemcitabine. Patients could continue on periodic maintenance with *BC-819* and gemcitabine, as long as they did not progress. Twelve patients have completed the first part of the study; 11/12 patients were evaluable for response. At their three-month assessment, 9/11 patients showed partial response (*n* = 2) or stable disease (*n* = 7). The adverse event profile in these patients was in line with that expected in a patient population with locally advanced pancreatic adenocarcinoma. All patients experienced AEs, mostly gastrointestinal or general disorders or laboratory abnormalities, primarily in liver function. No serious adverse event was considered by an investigator to be related to *BC-819*.

### 4.3. Ovarian Cancer

A Phase 1/2a dose escalation safety and preliminary efficacy study for the treatment of patients with advanced stage ovarian cancer has been completed. In addition, a patient with ovarian cancer was treated under a compassionate use protocol received sixteen IP infusions of *BC-819* at doses ranging from 80 mg to 140 mg of plasmid DNA per treatment for a total cumulative dose of 1.7 g; this was well-tolerated [47].

In the Phase 1/2a study, 14 subjects with recurrent, platinum resistant advanced stage ovarian cancer or primary peritoneal carcinoma were treated with intraperitoneally administered *BC-819*. These heavily pre-treated patients received escalating doses of 60*–*240 mg of *BC-819*. Again, the MTD was not reached. The majority of the AEs were laboratory abnormalities, general or gastrointestinal disorders mostly related to the patients’ general medical condition rather than to *BC-819*. The highest cumulative exposure by IP administration in this study was 720 mg to three subjects in the highest dose cohort (three administrations each of 240 mg). This dose was well-tolerated in all cases.

The best tumor response was stable disease; no patient demonstrated a complete or partial remission. As most patients had ascites, which often required paracentesis, standard assessment of response was problematic. A dose response effect on survival was observed, indicating an antitumor effect, though this should be interpreted with caution in the light of the small sample sizes and possible confounding variables among cohorts.

### 4.4. Colon Liver Metastases

It was shown in animal tumor models that the administration of *BC-819* is safe and efficacious in the hepatic cancer indication and preliminary evidence of tumor response was obtained in two compassionate use patients with hepatic metastases.

Two patients with colon liver metastases were treated during a named patient compassionate use program (one by intratumoral route of administration and one by hepatic artery infusion). The intratumoral injections were two doses of 6 mg each. The treatment was well tolerated, provided temporary palliative relief and showed radiographic evidence of a large area of tumor shrinkage. The second case was a patient with inoperable metastasis in both lobes of the liver. The patient received two courses of treatment each consisting of three infusions of *BC-819* via the common hepatic artery given once per week for three weeks, with the second course starting one month after the first infusion. The first course consisted of escalating doses of 16, 24 and 36 mg of *BC-819* DNA. The second course consisted of escalating doses of 48, 64 and 82 mg of *BC-819*. This patient had stable disease throughout the two months of treatment.

## 5. Conclusions and Future Perspectives

The emerging notion from the studies highlighted here is that the *H19* locus is much more complicated than previously thought. It houses genomic sequences that can transcribe giving various transcriptional outputs in both sense and antisense directions. This adds further complexity to the locus regulation within the context of cancer development and also imprinting maintenance. The *H19* locus, thus, is able to regulate biological processes on separate levels of significance through imprinting, production of *miR-675* and also a complex panel of expressions in both sense and antisense directions. However, the challenge consists of understanding the function of each transcriptional product separately and in relation to others and the function of the *H19* locus as a whole. Apparently, the controversy that exists when dealing with the *H19* function may be a reflection of its complexity.

It is also clear that in addition to cancer, *H19* RNA is linked to many other diseases. Although understanding the role of *H19* in tumorigenesis is the focus of many efforts in the past decade and has resulted in highlighting major findings regarding the regulation of this unique transcript, yet very little is known about the role of *H19* in other diseases and much more efforts should be invested in this direction.

Novel approaches to increase the cytotoxicity of the therapeutic plasmid and to increase the numbers of patients that can benefit from this therapy is currently under intensive investigation by our group. Moreover, the preclinical utility of short interfering RNA to knockdown *H19* RNA specifically is currently under development.

## References

[1] Cai X., Cullen B.R. (2007). The imprinted *H19* noncoding RNA is a primary microRNA precursor. RNA.

[2] Onyango P., Feinberg A.P. (2011). A nucleolar protein, *H19* opposite tumor suppressor (HOTS), is a tumor growth inhibitor encoded by a human imprinted *H19* antisense transcript. Proc. Natl. Acad. Sci. USA.

[3] Berteaux N., Aptel N., Cathala G., Genton C., Coll J., Daccache A., Spruyt N., Hondermarck H., Dugimont T., Curgy J.J. (2008). A novel *H19* antisense RNA overexpressed in breast cancer contributes to paternal IGF2 expression. Mol. Cell Biol.

[4] Court F., Baniol M., Hagege H., Petit J.S., Lelay-Taha M.N., Carbonell F., Weber M., Cathala G., Forne T. (2011). Long-range chromatin interactions at the mouse *IGF2*/*H19* locus reveal a novel paternally expressed long non-coding RNA. Nucleic Acids Res.

[5] Matouk I., Ayesh B., Schneider T., Ayesh S., Ohana P., de-Groot N., Hochberg A., Galun E. (2004). Oncofetal splice-pattern of the human *H19* gene. Biochem. Biophys. Res. Commun.

[6] Lin W.L., He X.B., Svensson K., Adam G., Li Y.M., Tang T.W., Paldi A., Pfeifer S., Ohlsson R. (1999). The genotype and epigenotype synergize to diversify the spatial pattern of expression of the imprinted *H19* gene. Mech. Dev.

[7] Okutsu T., Kuroiwa Y., Kagitani F., Kai M., Aisaka K., Tsutsumi O., Kaneko Y., Yokomori K., Surani M.A., Kohda T. (2000). Expression and imprinting status of human PEG8/IGF2AS, a paternally expressed antisense transcript from the IGF2 locus, in Wilms’ tumors. J. Biochem.

[8] Lee M.P., DeBaun M.R., Mitsuya K., Galonek H.L., Brandenburg S., Oshimura M., Feinberg A.P. (1999). Loss of imprinting of a paternally expressed transcript, with antisense orientation to KVLQT1, occurs frequently in Beckwith–Wiedemann syndrome and is independent of insulin-like growth factor II imprinting. Proc. Natl. Acad. Sci. USA.

[9] Smilinich N.J., Day C.D., Fitzpatrick G.V., Caldwell G.M., Lossie A.C., Cooper P.R., Smallwood A.C., Joyce J.A., Schofield P.N., Reik W. (1999). A maternally methylated CpG island in KvLQT1 is associated with an antisense paternal transcript and loss of imprinting in Beckwith–Wiedemann syndrome. Proc. Natl. Acad. Sci. USA.

[10] Smits G., Mungall A.J., Griffiths-Jones S., Smith P., Beury D., Matthews L., Rogers J., Pask A.J., Shaw G., VandeBerg J.L. (2008). Conservation of the *H19* noncoding RNA and *H19*-*IGF2* imprinting mechanism in therians. Nat. Genet.

[11] Keniry A., Oxley D., Monnier P., Kyba M., Dandolo L., Smits G., Reik W. (2012). The *H19* lincRNA is a developmental reservoir of miR-675 that suppresses growth and IGF1R. Nat. Cell Biol.

[12] Gao W.L., Liu M., Yang Y., Yang H., Liao Q., Bai Y., Li Y.X., Li D., Peng C., Wang Y.L. (2012). The imprinted *H19* gene regulates human placental trophoblast cell proliferation via encoding miR-675 that targets Nodal Modulator 1 (NOMO1). RNA Biol.

[13] Tsang W.P., Ng E.K., Ng S.S., Jin H., Yu J., Sung J.J., Kwok T.T. (2010). Oncofetal *H19*-derived miR-675 regulates tumor suppressor RB in human colorectal cancer. Carcinogenesis.

[14] Dudek K.A., Lafont J.E., Martinez-Sanchez A., Murphy C.L. (2010). Type II collagen expression is regulated by tissue-specific miR-675 in human articular chondrocytes. J. Biol. Chem.

[15] Matouk I., Ohana P., Ayesh S., Hochberg A. (2005). The oncofetal *H19* RNA in human cancer, from the bench to the patient. Cancer Ther.

[16] Matouk I.J., Ohana P., Galun E., Hochberg A (2008). The Pivotal Role of the Oncofetal *H19* RNA in Human Cancer, A New Hope. Gene Therapy and Cancer Research Focus.

[17] Matouk I.J., deGroot N., Mezan S., Ayesh S., Abu-Lail R., Hochberg A., Galun E. (2007). The *H19* non-coding RNA is essential for human tumor growth. PLoS One.

[18] Matouk I.J., Mezan S., Mizrahi A., Ohana P., Abu-Lail R., Galun E., Hochberg A. (2010). The oncofetal *H19* RNA connection: Hypoxia, p53 and cancer. Biochim. Biophys. Acta.

[19] Barsyte-Lovejoy D., Lau S.K., Boutros P.C., Khosravi F., Jurisica I., Andrulis I.L., Tsao M.S., Penn L.Z. (2006). The *c-Myc* oncogene directly induces the *H19* noncoding RNA by allele-specific binding to potentiate tumorigenesis. Cancer Res.

[20] Ayesh S., Matouk I., Schneider T., Ohana P., Laster M., Hochberg A. (2002). Possible physiological role of *H19* RNA. Mol. Carcinog.

[21] Berteaux N., Lottin S., Monté D., Pinte S., Quatannens B., Coll J., Hondermarck H., Curgy J.J., Dugimont T., Adriaenssens E. (2005). *H19* mRNA-like noncoding RNA promotes breast cancer cell proliferation through positive control by E2F1. J. Biol. Chem.

[22] Dugimont T., Montpellier C., Adriaenssens E., Lottin S., Dumont L., Iotsova V., Lagrou C., Stéhelin D., Coll J., Curgy J.J. (1998). The *H19* TATA-less promoter is efficiently repressed by the wild- type tumor suppressor gene product p53. Oncogene.

[23] Choufani S., Shuman C., Weksberg R. (2010). Beckwith–Wiedemann syndrome. Am. J. Med. Genet. C.

[24] Joyce J.A., Lam W.K., Catchpoole D.J., Jenks P., Reik W., Maher E.R., Schofield P.N. (1997). Imprinting of IGF2 and *H19*: Lack of reciprocity in sporadic Beckwith–Wiedemann Syndrome. Hum. Mol. Genet.

[25] Eggermann T., Begemann M., Binder G., Spengler S. (2010). Silver-Russell syndrome: Genetic basis and molecular genetic testing. Orphanet. J. Rare Dis.

[26] Gabory A., Ripoche M.A., le Digarcher A., Watrin F., Ziyyat A., Forné T., Jammes H., Ainscough J.F., Surani M.A., Journot L., Dandolo L. (2009). *H19* acts as a trans regulator of the imprinted gene network controlling growth in mice. Development.

[27] Schonherr N., Binder G., Korsch E., Kammerer E., Wollmann H.A., Eggermann T. (2008). Are *H19* variants associated with Silver-Russell syndrome?. J. Pediatr. Endocrinol. Metab.

[28] Perkins E., Murphy S.K., Murtha A.P., Schildkraut J., Jirtle R.L., Demark-Wahnefried W., Forman M.R., Kurtzberg J., Overcash F., Huang Z., Hoyo C. (2012). Insulin-like growth factor 2/*H19* methylation at birth and risk of overweight and obesity in children. J. Pediatr.

[29] Heijmans B.T., Tobi E.W., Stein A.D., Putter H., Blauw G.J., Susser E.S., Slagboom P.E., Lumey L.H. (2008). Persistent epigenetic differences associated with prenatal exposure to famine in humans. Proc. Natl. Acad. Sci. USA.

[30] Grivennikov S.I., Greten F.R., Karin M. (2010). Immunity, inflammation, and cancer. Cell.

[31] Stuhlmüller B., Kunisch E., Franz J., Martinez-Gamboa L., Hernandez M.M., Pruss A., Ulbrich N., Erdmann V.A., Burmester G.R., Kinne R.W. (2003). Detection of oncofetal *H19* RNA in rheumatoid arthritis synovial tissue. Am. J. Pathol.

[32] Smitten A.L., Simon T.A., Hochberg M.C., Suissa S. (2008). A meta-analysis of the incidence of malignancy in adult patients with rheumatoid arthritis. Arthritis Res. Ther.

[33] Love T., Solomon D.H. (2008). The relationship between cancer and rheumatoid arthritis: Still a large research agenda. Arthritis Res. Ther.

[34] Bergamaschi S., Morato E., Bazzo M., Neves F., Fialho S., Castro G., Zimmermann A., Pereira I. (2012). Tumor markers are elevated in patients with rheumatoid arthritis and do not indicate presence of cancer. Int. J. Rheum. Dis.

[35] Carty D.M., Delles C., Dominiczak A.F. (2008). Novel biomarkers for predicting preeclampsia. Trends Cardiovasc. Med.

[36] Eggenschwiler J., Ludwig T., Fisher P., Leighton P.A., Tilghman S.M., Efstratiadis A. (1997). Mouse mutant embryos overexpressing IGF-II exhibit phenotypic features of the Beckwith–Wiedemann and Simpson-Golabi-Behmel syndromes. Genes Dev.

[37] Yu L., Chen M., Zhao D., Yi P., Lu L., Han J., Zheng X., Zhou Y., Li L. (2009). The *H19* gene imprinting in normal pregnancy and pre-eclampsia. Placenta.

[38] Vaiman D., Mondon F., Garcès-Duran A., Mignot T.M., Robert B., Rebourcet R., Jammes H., Chelbi S.T., Quetin F., Marceau G. (2005). Hypoxia-activated genes from early placenta are elevated in Preeclampsia, but not in Intra-Uterine Growth Retardation. BMC Genomics.

[39] Bourque D.K., Avila L., Peñaherrera M., von Dadelszen P., Robinson W.P. (2010). Decreased placental methylation at the *H19/IGF2* imprinting control region is associated with normotensive intrauterine growth restriction but not preeclampsia. Placenta.

[40] Gao W.L., Li D., Xiao Z.X., Liao Q.P., Yang H.X., Li Y.X., Ji L., Wang Y.L. (2011). Detection of global DNA methylation and paternally imprinted *H19* gene methylation in preeclamptic placentas. Hypertens Res.

[41] Rachmilewitz J., Gileadi O., Eldar-Geva T., Schneider T., De-Groot N., Hochberg A. (1992). Transcription of the *H19* gene in differentiating cytotrophoblasts from human placenta. Mol. Reprod. Dev.

[42] Adam G.I., Cui H., Miller S.J., Flam F., Ohlsson R. (1996). Allele-specific *in situ* hybridization (ASISH) analysis: A novel technique which resolves differential allelic usage of *H19* within the same cell lineage during human placental development. Development.

[43] Walsh C., Miller S.J., Flam F., Fisher R.A., Ohlsson R. (1995). Paternally derived *H19* is differentially expressed in malignant and nonmalignant trophoblast. Cancer Res.

[44] Smets E.M., Visser A., Go A.T., van Vugt J.M., Oudejans C.B. (2006). Novel biomarkers in preeclampsia. Clin. Chim. Acta.

[45] Sidi A., Ohana P., Benjamin S., Shalev M., Ransom J.H., Lamm D., Hochberg A., Leibovitch I. (2008). Phase I/II marker lesion study of intravesical BC-819 DNA plasmid in *H19* overexpressing superficial bladder cancer refractory to *Bacillus* calmette guerin. J. Urology.

[46] Hanna N., Ohana P., Konikoff F.M., Leichtmann G., Hubert A., Appelbaum L., Kopelman Y., Czerniak A., Hochberg A. (2012). Phase 1/2a, dose-escalation, safety, pharmacokinetic, and preliminary efficacy study of intratumoral administration of BC-819 in patients with unresectable pancreatic vancer. Cancer Gene Ther.

[47] Mizrahi A., Czerniak A., Ohana P., Amiur S., Gallula J., Matouk I., Abu-lail R., Birman T., Hochberg A., Levy T. (2010). Treatment of ovarian cancer ascites by intra-peritoneal injection of diphtheria toxin A chain-*H19* vector: A case report. J. Med. Case Rep.

